# Sex differences in pain catastrophizing and its relation to the transition from acute pain to chronic pain

**DOI:** 10.1186/s12871-024-02496-8

**Published:** 2024-04-02

**Authors:** Linh H.L. Le, Vanessa A.V. Brown, Sander Mol, Kaoutar Azijli, Martijn M. Kuijper, Leonie Becker, Seppe S.H.A. Koopman

**Affiliations:** 1 Erasmus University Rotterdam, Rotterdam, The Netherlands; 2 Department of Emergency Medicine, Franciscus Gasthuis and Vlietland, Rotterdam, The Netherlands; 3 Department of Emergency Medicine, Amsterdam University Medical Center, Amsterdam, The Netherlands; 4 Maasstad Academy, Maasstad Hospital, Rotterdam, The Netherlands; 5 Department of Cardiology, Maasstad Hospital, Rotterdam, The Netherlands; 6 Department of Anaesthesiology, Maasstad Hospital, Maasstadweg 21 3079 DZ Rotterdam, Rotterdam, The Netherlands

**Keywords:** Pain chronification, Pain catastrophizing, Differences between sexes

## Abstract

**Background and importance:**

Differences exist between sexes in pain and pain-related outcomes, such as development of chronic pain. Previous studies suggested a higher risk for pain chronification in female patients. Furthermore, pain catastrophizing is an important risk factor for chronification of pain. However, it is unclear whether sex differences in catastrophic thinking could explain the sex differences in pain chronification.

**Objectives:**

The aim of this study was to examine sex differences in pain catastrophizing. Additionally, we investigated pain catastrophizing as a potential mediator of sex differences in the transition of acute to chronic pain.

**Design, settings and participants:**

Adults visiting one of the 15 participating emergency departments in the Netherlands with acute pain-related complaints. Subjects had to meet inclusion criteria and complete questionnaires about their health and pain.

**Outcomes measure and analysis:**

The outcomes in this prospective cohort study were pain catastrophizing (short form pain catastrophizing) and pain chronification at 90 days (Numeric Rating Scale ≥ 1). Data was analysed using univariate and multivariable logistic regression models. Finally, stratified regression analyses were conducted to assess whether differences in pain catastrophizing accounted for observed differences in pain chronification between sexes.

**Main results:**

In total 1,906 patients were included. Females catastrophized pain significantly more than males (*p* < 0.001). Multiple regression analyses suggested that pain catastrophizing is associated with pain chronification in both sexes.

**Conclusions:**

This study reported differences between sexes in catastrophic cognitions in the development of chronic pain. This is possibly of clinical importance to identify high-risk patients and ensure an early intervention to prevent the transition from acute to chronic pain.

**Supplementary Information:**

The online version contains supplementary material available at 10.1186/s12871-024-02496-8.

## Introduction

Pain is one of the most common complaints in emergency departments (ED) [9]. Even though 70–90% of patients visiting the ED complain of pain [2], undertreatment remains a problem [4]. Undertreatment increases the risk of developing chronic pain [19]. Many definitions of chronic pain have been proposed, one of which is pain persisting beyond three months [30]. Chronic pain forms an enormous burden on health care in the Netherlands with an overall prevalence of 18% for moderate to severe pain in 2010 [1]. Chronification of pain has many consequences, such as decreased quality of life, overutilization of healthcare, loss of productivity and possibly opioid dependency [18].

Multiple pre-hospital risk factors for development of chronic pain have been identified. These include older age, female sex, pain catastrophizing, high-intensity acute pain, less than college education, low socio-economic status, anxiety, and depression [3]. Pain catastrophizing is an emotional and cognitive response to pain and is comprised of a tendency to ruminate, magnify, or feel helpless [16]. Previous studies found that pain catastrophizing contributed to a higher probability of developing chronic pain [3; 8; 10; 12; 19]. Besides an individual association with pain chronification, interactions between these risk factors exist as well. For example, studies have shown that pain catastrophizing interacts with depression, pain intensity, age, level of education, employment status, alcohol dependency, smoking, satisfaction with care received and marital status/relationship [6–8; 10; 24].

As of yet, the direct relationship between pain catastrophizing, sex, and pain chronification is unknown. Previous studies showed that females are more at risk for developing chronic pain [3; 17; 19]. Differences between sexes also exist in pain intensity [8; 12; 13; 16; 17]. This could imply that there are sex differences in the way pain is catastrophized. Previous clinical and experimental studies have been inconsistent about this [7; 8; 12; 13; 16; 22; 24]. Some studies suggested that catastrophizing cognitions or coping strategies were more frequent in females [8; 12; 13; 24]. This may suggest that pain catastrophizing is a potential intermediate in sex differences in occurrence of pain and its chronification. However, other studies concluded no significant differences between sexes in pain catastrophizing [7; 16; 22]. Furthermore, previous studies only determined the association between pain catastrophizing and sex in specific patient groups. They only investigated certain pain causes, such as osteoarthritis, musculoskeletal injury, or motor vehicle accidents with acute whiplash injury [3; 12; 13; 19; 22]. Also, they only included patients with specific locations of pain such as neck, shoulders, lower back, or knee pain [6; 10; 12; 13; 22].

An intervention on pain catastrophizing could be used to prevent the transition from acute to chronic pain. The four-item short form of the pain catastrophizing scale (PCS) could be considered as a screening variable to identify high-risk patients, since it is brief and accessible. In the 13-item PCS a score of 30 or more indicates a high level of catastrophizing, which is clinically relevant [25]. As far as we know, no cut-off score for the four-item short form of the PCS has been determined yet. Pain catastrophizing could also be applied as a target for intervention and treatment in an early stage of pain. Earlier studies with cognitive behavioural interventions have found improvements in pain and disability with reduction in pain catastrophizing [20].

To our knowledge, the relationship between pain catastrophizing, sex, and development of chronic pain within all patients presenting with pain in the ED has not been studied yet. The primary aim is to study the potential differences in pain catastrophizing between sexes. Our second aim is to study the relationship between pain catastrophizing, sex, and pain chronification in all patients presenting in the ED with a pain related problem who are discharged the same day. Our hypothesis is that sex differences in the risk of developing chronic pain are (partly) explained by the sex differences in pain catastrophizing.

## Methods

### Design and subjects

This article is a substudy of the PRACTICE study with the aim to study the relationship between sex, pain catastrophizing and pain chronification. For this study, data from the PRACTICE study was used. The PRACTICE study is a prospective, multicentre, longitudinal study aimed at developing a prediction model for patients at risk of developing chronic pain. The full description of design, subjects, and procedure of this study can be found in the study of Ten Doesschate, et al. [26]. This study was conducted between August 2018 and April 2020 in 15 EDs in the Netherlands including hospitals of all types. The study population was representative for the Dutch population regarding injury, age and sex. Data were collected with questionnaires about health, quality of life, and pain with a total follow-up of 180 days. Patients of 18 years and older were included when visiting the ED for an acute pain related cause and discharged without admission. Only patients without admission were included because we were interested in studying these patients exclusively.

Exclusion criteria were cognitive impairment, illiteracy, a language barrier, a current diagnosis of chronic pain located at or near the location of their current complaint, a hospital admission or acute pain within seven days after surgery.

### Ethic approval and consent to participate

The Medical research ethics committee (METC, Protocol 2018-39) approved the study. Local approval was obtained by all participating centres and was conducted in accordance to the principles of the Declaration of Helsinki. Patients provided written informed consent according to the procedure approved by the METC.

### Procedure

All consecutive patients presenting at the emergency department with an acute pain-related complaint were asked for participation if meeting the in- and exclusion criteria. Patients were recruited consecutively as they presented to the ED. During the first month of the study, patients received questionnaires on paper. During the rest of the study, patients received questionnaires in a web-based electronic application. Paper questionnaires were collected the first month to validate the electronic application. The study protocol for both groups were equal. In the emergency department, patients received usual care without additional interventions.

### Outcome measures

Baseline characteristics were collected from electronic patient records. These include age, sex, date and time of arrival and discharge, treatment time, triage priority, numeric rating scale (NRS) of pain on arrival at the ED (NRS0), location and cause of pain, type of injury, pain-management and follow-up. Other variables (e.g. pain catastrophizing, pain lasting more than 90 days (NRS90) were collected from questionnaires.

During seven consecutive days after discharge patients were asked daily for their NRS, the use of painkillers (and specification of the used painkillers) and extra visits. Furthermore, patients were queried during these days about depression and treatment, whether or not patients are in a relationship, pre-existing chronic pain, alcohol consumption, education, employment and sick leave, smoking and satisfaction with emergency department care (supplemental Table 1). Education level was categorised in low, intermediate or high. Low level of education: primary school, Pre-vocational secondary education, Secondary vocational education level 1 Or completion of the first three years of Senior general secondary education or Pre-university education. Intermediate level of education: graduation on senior general secondary education, pre-university education, secondary vocational education level 2–4. High level of education: Graduation at least university of applied sciences.

On the fifth and sixth day patients received the four-item short form of the PCS, which measures the level of pain catastrophizing [5; 15]. It is a five-point self-report scale indicating the degree to which participants experience certain thoughts or feelings when having pain (0 = not at all, 4 = all the time). A higher score indicates more catastrophic thinking. On the seventh day after discharge, they also received the Euroqol five-dimension five-level (EQ-5D-5 L) questionnaire. The NRS, the EQ-5D-5 L questionnaire, question 7 and 8 of the 36-item Short Form Survey (SF-36) and the Brief Pain Inventory (BPI)were asked at day 90 and 180.

Outcomes were PCS and pain chronification. The development of chronic pain was based on dichotomisation of reported severity of pain on day 90, in which NRS = 0 was defined as no chronic pain, and NRS ≥ 1 as chronic pain.

### Statistical analysis

Descriptive statistics were reported as frequency (%) for categorical data and mean ± standard deviation (SD) or median with interquartile range (IQR, 25th-75th percentile) for continuous data.

All relevant questionnaires were examined for missing data. Missing data was imputed using multiple imputation by chained equations (MICE) [29] with outcome and baseline variables (sex, age, NRS0, pain location, trauma, fracture, satisfaction with care received, depression and treatment, relationship, pre-existing chronic pain, alcohol consumption, education, employment and sick leave, smoking, PCS, NRS90, and pain chronification) in the imputation model to create 100 imputed data sets. Imputation was only done after testing that data was missing at random. Supplemental Table 2 gives a complete overview of the imputed variables.

The PCS of patients with and without pain chronification were compared. Univariate and multivariable logistic regression models were performed on imputed datasets to correct for possible confounders while studying sex differences in pain catastrophizing and the risk of developing chronic pain. Baseline variables were tested as possible confounders. A chi-squared test was conducted for categorical data. Wilcoxon-Mann-Whitney test was conducted for numerical, non-normally distributed data. Normally distributed numerical data was compared using the student’s t-tests.

A regression analysis studying the relation between PCS and chronic pain was performed corrected for sex and other confounders. Tested variables were chosen based on previous literature, clinical reasoning or identified through regression analysis. Logistic regression analysis with interaction terms were performed to identify effect modifiers. Potential confounders were chosen based on a model built on previous literature, clinical reasoning, clinical experience and by drawing a causal directed acyclic graph (DAG) (Supplemental Fig. 1) [27]. Based on the DAG, the algorithm selects variables for which needs to be corrected to allow for an estimation of the causal effect of the exposure. Data was presented as odds ratio (OR) with 95% confidence intervals (95% CI). Finally, stratified regression analyses were conducted to assess whether differences in pain catastrophizing accounted for observed differences in pain chronification between sexes. This in order to exclude effect modification by gender.

Sensitivity analyses were conducted with different definitions of pain chronification. These include any pain (NRS ≥ 1) and moderate to severe pain (NRS ≥ 4) lasting more than 90 days [1; 19]. These cut-offs represent different definitions of chronic pain used in literature. Baseline characteristics were compared between responders and non-responders (missing data) to check whether data was missing-at-random. In all analyses, a p-value < 0.05 was considered statistically significant. The data were analyzed using R version 4.0.2 [21].

## Results

**Fig. 1:**
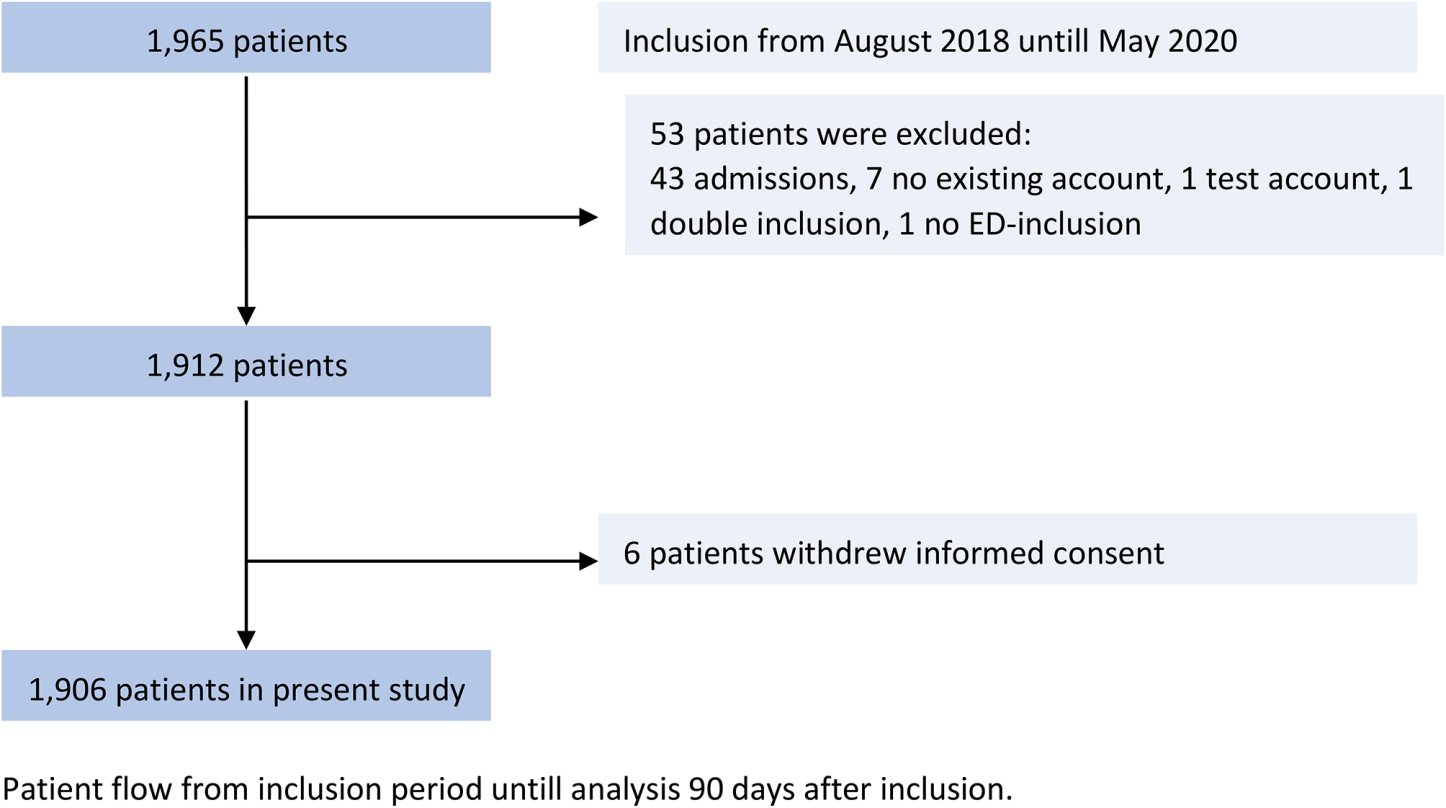
Flowchart

In total, 1965 patients were included of which 1906 patient remained after in- and exclusion criteria were applied. Of the 1,906 analysed patients, 6 participants (0.3%) had missing sex data. 1,009 participants (52.9%) responded to the four-item short form of the PCS, and 825 participants (43.3%) returned the questionnaires on pain after 90 days (Fig. 1). Missing data for other variables ranged from 0.3 to 46.7%. Baseline patient characteristics differed significantly between responders and non-responders on several variables. Patients in the responder group were older (47 vs. 45) and more often female (53.1% females vs. 45.9% males). Responders visited the emergency department more often with fractures (57.9% vs. 48.4%) and were more often non-smokers (12.7% vs. 24.3%). Patients in the non-responder group had more comorbidities (55.3% vs. 48.0%).

**Fig. 2:**
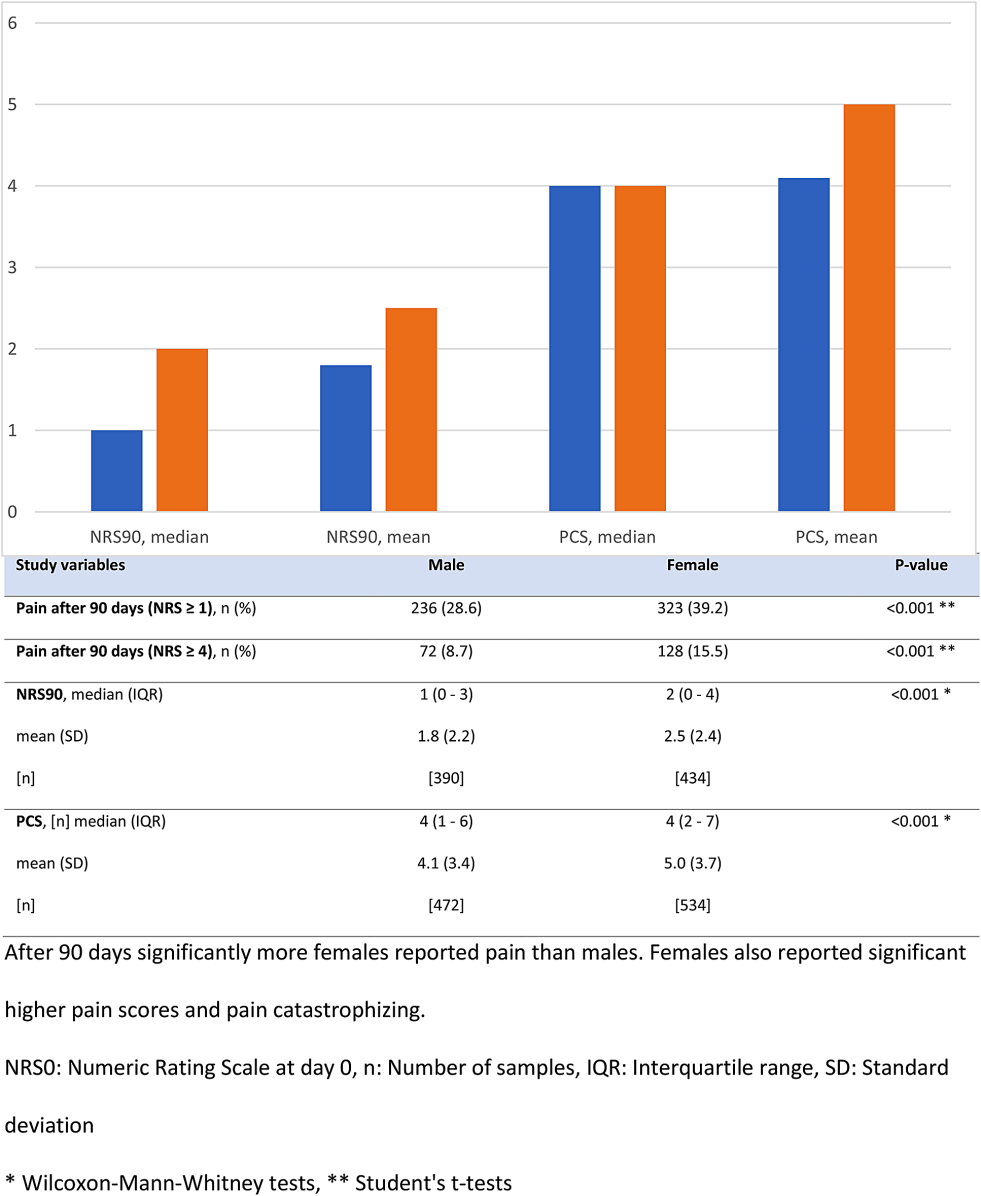
Pain scores at 90 days and pain catastrophizing per gender

A description of baseline characteristics is provided in Table 1. Significantly more males had pain in upper extremities (27.2% vs. 21.9%, *p* < 0.001) while females had more pain in the lower extremities (15.6% vs. 18.9%, *p* < 0.001). There were no significant differences found between males and females in employment, level of education, and relationship status. Univariately, females had on average a higher PCS compared to males, although the median PCS was similar (Fig. 2). Regardless of the definition used, significantly more females developed chronic pain than males. The incidence of chronic pain did not differ between participating centres (*p* = 0.339).

The role of pain catastrophizing on pain chronification was examined using both a regression analyses corrected for sex as a stratified regression analyses by sex (Tables 2 and 3). The odds ratio reported here are per step on the pain catastrophizing scale (4-step scale, 0 = no pain catastrophizing, 0 = reference category). A significant association was found after correction for confounders between pain chronification and pain catastrophizing (OR 1.17; 95% CI: 1.05–1.29; *P* < 0.01). Using the alternative definition for pain chronification (NRS ≥ 4), we only found a significant association for the female group (OR 1.11; 95% CI: 1.03–1.20; *P* < 0.01). Several confounders, such as age, were entered in this stratified regression analyses (Table 3). Age was significantly associated with chronic pain development irrespective of sex or outcome definition used. Education was significantly associated with pain chronification if NRS ≥ 1 was used as outcome. Stratified analyses showed no indication of effect modification by sex (Table 3).

**Table 1:** Baseline characteristics for male and female participants

Study variables	Male	Female	P-value
Total, n (%)	973 (51.1)	925 (48.7)	0.120
Age, median (IQR)	41 (27–55)	49.0 (33–62)	< 0.001 *
mean (SD)	41.7 (16.8)	48.2 (17.6)	
[n]	[968]	[920]	
NRS0, median (IQR)	5.0 (2–7)	6.0 (3–7)	< 0.001 *
mean (SD)	4.6 (2.6)	5.2 (2.6)	
[n]	[972]	[922]	
Trauma, n (%)	569 (44.5)	529 (41.4)	0.567 **
Fracture, n (%)	454 (23.9)	529 (27.8)	< 0.001 **
Satisfaction with care received, median (IQR)	8 (7–9)	8 (7–9)	0.032 *
mean (SD)	7.8 (1.7)	7.6 (1.7)	0.090
[n]	[480]	[533]	
**Depression, n (%)**	64 (5.6)	153 (13.3)	< 0.001 **
Treatment, n (%)	18 (8.2)	43 (19.6)	0.823 **
Chronic pain in other location, n (%)	94 (8.5)	158 (14.3)	< 0.001 **
**Alcohol consumption, n (%)**	298 (26.7)	239 (21.4)	< 0.001 **
Consumption per week, median (IQR)	5 (2–8)	4 (2–7)	< 0.001 *
mean (SD)	7.2 (11.1)	4.7 (3.9)	
[n]	[298]	[239]	
Smoking, n (%)	99 (9.7)	64 (6.3)	< 0.001 **

Significant differences were found between sexes in age, NRS0, fractures, satisfaction with treatment, depression, chronic pain in other locations, alcohol consumption, and smoking

NRS: Verbal Numeric Rating Scale, NRS90: (Verbal) Numeric Rating Scale at day 90, PCS: Pain Catastrophizing Scale, n: Number of samples, IQR: Interquartile range, SD: Standard deviation

* Wilcoxon-Mann-Whitney tests, ** Student’s t-tests

**Table 2:** Regression analysis on the association between pain catastrophizing and pain chronification corrected for gender

Pain chronification (NRS ≧ 1 at day 90)				
	Odds ratio	95% CI		P-value
PCS	**1.13**	**1.05**	**1.21**	**0.001**
Gender	1.62	1.05	2.48	0.028
Age	1.02	1.01	1.04	0.006
Depression	1.00	0.54	1.84	0.999
Pre-existent chronic pain	1.52	0.86	2.68	0.149
Alcohol consumption	0.69	0.43	1.10	0.122
Low education	REF0	REF	REF	REF
Intermediate education	0.14	0.04	0.44	0.001
High education	0.25	0.08	0.82	0.025
Smoking	1.07	0.53	2.14	0.853
**Pain chronification (NRS 4 at day 90)**				
PCS	**1.10**	**1.04**	**1.18**	**0.002**
Gender	1.52	0.98	2.36	0.064
Age	1.02	1.01	1.04	0.004
Depression	0.74	0.40	1.38	0.344
Pre-existent chronic pain	1.73	0.99	3.00	0.054
Alcohol consumption	0.87	0.58	1.31	0.509
Low education	REF0	REF	REF	REF
Intermediate education	0.34	0.09	1.26	0.113
High education	0.57	0.14	2.34	0.439
Smoking	1.21	0.64	2.29	0.555

Pain catastrophizing was significantly associated with chronification of pain. We corrected for multiple possible confounders

Education level was self-reported by the patient. Low level of education: primary school, Pre-vocational secondary education, Secondary vocational education level 1 Or completion of the first three years of Senior general secondary education or Pre-university education

Intermediate level of education: graduation on senior general secondary education, pre-university education, secondary vocational education level 2-4

High level of education: Graduation at least university of applied sciences

A chi-squared test was conducted for categorical data. Wilcoxon-Mann-Whitney test was conducted for numerical, non-normally distributed data

NRS: Numeric Rating Scale, PCS: Pain Catastrophizing Scale, CI: Confidence interval

**Table 3:** Multiple logistic regression analysis on the association between PCS and pain chronification, stratified by sex and corrected for potential confounders

	Pain chronification (NRS 1 at day 90)									
	**Male**					**Female**				
	Odds ratio	95% CI			P-value	Odds ratio	95% CI		P-value	
PCS	**1.17**	**1.05**	**1.29**	**0.004**		**1.11**	**1.01**	**1.21**		**0.024**
Age	1.02	1.00	1.04	0.037		1.02	1.00	1.04		0.020
Depression	1.08	0.42	2.79	0.869		0.96	0.47	1.96		0.920
Pre-existent chronic pain	1.52	0.68	3.39	0.307		1.52	0.80	2.92		0.204
Alcohol consumption	0.80	0.44	1.47	0.475		0.61	0.34	1.09		0.099
Low education	REF	REF	REF	REF		REF	REF	REF		REF
Intermediate education	0.14	0.04	0.55	0.006		0.11	0.03	0.47		0.004
High education	0.27	0.07	0.99	0.053		0.20	0.05	0.86		0.035
Smoking	1.07	0.46	2.50	0.877		1.09	0.41	2.91		0.867
	**Pain chronification (NRS 4 at day 90)**									
PCS	1.09	0.99	1.20	0.083		**1.11**	**1.03**	**1.20**		**0.006**
Age	1.03	1.01	1.05	0.013		1.02	1.00	1.04		0.025
Depression	0.68	0.24	1.90	0.460		0.78	0.39	1.52		0.461
Pre-existent chronic pain	2.03	0.89	4.64	0.095		1.57	0.85	2.90		0.150
Alcohol consumption	0.84	0.45	1.58	0.587		0.90	0.55	1.47		0.666
Low education	REF0	REF	REF	REF		REF	REF	REF		REF
Intermediate education	0.28	0.06	1.35	0.119		0.37	0.09	1.48		0.166
High education	0.59	0.13	2.67	0.499		0.54	0.12	2.54		0.444
Smoking	1.53	0.68	3.47	0.308		0.92	0.39	2.22		0.860

A significant correlation was found between pain chronification and pain catastrophizing for both sexes if NRS. A similar effect of pain catastrophizing on pain chronification between sexes was shown. The odds ratio reported here are per step on the pain catastrophizing scale (4-step scale, 0 = no pain catastrophizing, 0 = reference category)

Education level was self-reported by the patient. Low level of education: primary school, Pre-vocational secondary education, Secondary vocational education level 1 Or completion of the first three years of Senior general secondary education or Pre-university education

Intermediate level of education: graduation on senior general secondary education, pre-university education, secondary vocational education level 2-4

High level of education: Graduation at least university of applied sciences

A chi-squared test was conducted for categorical data. Wilcoxon-Mann-Whitney test was conducted for numerical, non-normally distributed data

NRS: Numeric Rating Scale, PCS: Pain Catastrophizing Scale, CI: Confidence interval

## Discussion

In this study, we studied potential differences in pain catastrophizing between sexes. We showed that females catastrophize pain more often than males. Furthermore, our data showed that pain catastrophizing increased the risk of chronic pain in both males and females when chronic pain was defined as an NRS ≧ 1 at 90 days. When chronic pain was defined as an NRS ≧ 4 at 90 days, pain catastrophizing was associated with chronic pain in females. In males, the association was not statistically significant. To our knowledge, this was the first study investigating the relationship between pain catastrophizing, sex, and pain chronification in patients presenting in an emergency department with any cause of pain. Pain catastrophizing increased the risk of pain chronification, irrespective of sex. An intervention to reduce pain catastrophizing might thus reduce the risk of pain chronification, although this cannot be concluded based on our research.

Our finding that females tend to catastrophize more was consistent with previous studies. These studies showed that females reported higher levels of catastrophic thinking in both healthy people and chronic pain patients [7, 8, 12, 13]. Females reported more pain, a higher pain intensity, more frequent and longer episodes of pain, poorer pain-related outcomes, and lower pain tolerance [7, 8, 28]. They used more emotion-based coping strategies whereas males used more problem focused ones [13]. There were sex differences in pain due to socialization, social and cultural norms, and expectations regarding social roles [28]. For example, males are expected to be stoic, minimizing, and enduring pain, which could lead to underreporting of pain and catastrophizing by males [28]. Females reported pain sooner to reduce its impact as they were often fulfilling more roles, like taking care of children or elderly people, household, and work [28]. 

We have shown a statistically significant relationship between pain catastrophizing and pain chronification (NRS ≧ 1) irrespective of sex. If chronic pain was defined as NRS at 90 days ≧ 4, it was only statistically significant in females. Our results were partly consistent with earlier experimental and clinical studies in specific populations [7; 12; 13; 24]. Pierik, et al. stated that patients who catastrophized their pain were three times more prone for transition into chronic pain [19]. Multiple studies have also shown that catastrophizing (partly) mediated for other pain-related outcomes, such as pain intensity, pain tolerance, and pain disability [6; 7, 12, 16, 24].

Although our data suggests a differential relationship between pain catastrophizing, chronic pain and sex depending on the cut-off point for chronic pain (NRS ≧ 1 vs. NRS ≧ 4), this might be due to a lack of power. This might be due to several limitations. Firstly, despite reminders, 48.3% of participants did not respond to all four questions about pain catastrophizing and 56.8% did not report their NRS after 90 days. This could limit the strength and representativeness of the results. This low response rate might explain the lack of association of depression and smoking with chronic pain that previous studies have reported [12; 24]. We imputed data to reduce the chance of a type II error.

Our results showed that 28.4% of males and 39.3% of females had chronic pain, which seems unlikely compared to the 18% prevalence previously stated in other studies. This could be explained by different definitions of chronic pain. Chronic pain in our study was defined as any pain lasting more than 90 days (NRS ≥ 1). Other studies defined chronic pain as moderate to severe pain (NRS ≥ 4) [1; 19]. Using the latter definition, 8.5% and 15.6% (males and females respectively) developed chronic pain. Our analysis showed similar results for different definitions of pain chronification, which strengthens our findings.

In addition, all participants participated voluntarily. Participants who completed questionnaires might differ from patients refusing participation, patients lost to follow up or quitting the study. Unfortunately, we could not compare baseline characteristics between patients who provided informed consent and those who did not, since no consent was given for collecting data from patient registries. Baseline characteristics of patients with and without missing data were mostly comparable (Supplemental Table 3). Furthermore, patients free from pain might not feel the need to report their NRS90. A catastrophic mindset could also lead to more willingness to participate and report, which could have led to overestimation. This selection bias is a common problem in studies requiring volunteers [12].

Finally, the relationship between variables, such as pain intensity, satisfaction with care received, and catastrophizing could be confounded by cause and treatment of the underlying affliction. In this study cause and pain characteristics (for example nociceptive, neuropathic) were not included in the analysis due to the large number of variables that were already examined. Many variables should be considered, such as causes, comorbidity, type and time of intervention and/or medications, and whether patients followed given advice. Treatment by attending physician or location is possible but unlikely given the absence of differences in incidence rate of chronic pain between different locations.

Despite the limitations, our study has several strengths. This study showed results that have important clinical implications for pain treatment in the acute setting. Furthermore, we conducted a study in patients with any cause or severity of pain, which makes it more likely that these findings can be generalized.

In conclusion, this study confirms the sex differences in pain catastrophizing in patients visiting the ED for pain-related complaints. Our data suggested that pain catastrophizing increased the risk of pain chronification, irrespective of sex. This study could be relevant for the assessment and management of acute pain in the ED to prevent transition into chronic pain. High-risk patients, namely those who catastrophize their pain, could be detected, and pain chronification might be prevented. Whether reducing pain catastrophizing indeed leads to less pain chronification is a topic in need of further studying.

* Inclusion sites.

- Albert Schweitzer Hospital location Dordwijk, Dordrecht.
- Albert Schweitzer Hospital, Zwijndrecht.
- Amsterdam University Medical Center location AMC, Amsterdam.
- Amsterdam University Medical Center location VU, Amsterda.
- Catharina hospital, Eindhoven.
- Erasmus Medical Center, Rotterdam.
- Franciscus Hospital Location Gasthuis & Vlietland, Rotterdam & Schiedam.
- Haaglanden Medical Center – Bronovo, Den Haag.
- Haaglanden Medical Center – Westeinde, Den Haag.
- Leiden University Medical Center, Leiden.
- Maasstad Hospital, Rotterdam.
- Reinier de Graaf Hospital, Delft.
- Zuyderland Medical Center- Heerlen.
- Zuyderland Medical Center- Sittard-Geleen.

### Electronic supplementary material

Below is the link to the electronic supplementary material.

Supplementary Material 1

Supplementary Material 2

Supplementary Material 3

Supplementary Material 4

## Data Availability

Data is available upon reasonable request to the corresponding author (koopmanj@maasstadziekenhuis.nl).
