# Supplementary material for: Sex differences in pain catastrophizing and its relation to the transition from acute pain to chronic pain
Source: BMC Anesthesiol. 2024 Apr 2;24:127. doi: 10.1186/s12871-024-02496-8 (PMC10985981; doi:10.1186/s12871-024-02496-8)
Supplement: Supplementary file 1 — Supplementary Material 1 [file 12871_2024_2496_MOESM1_ESM.docx]

Supplemental figure 1: Causal directed acyclic graph


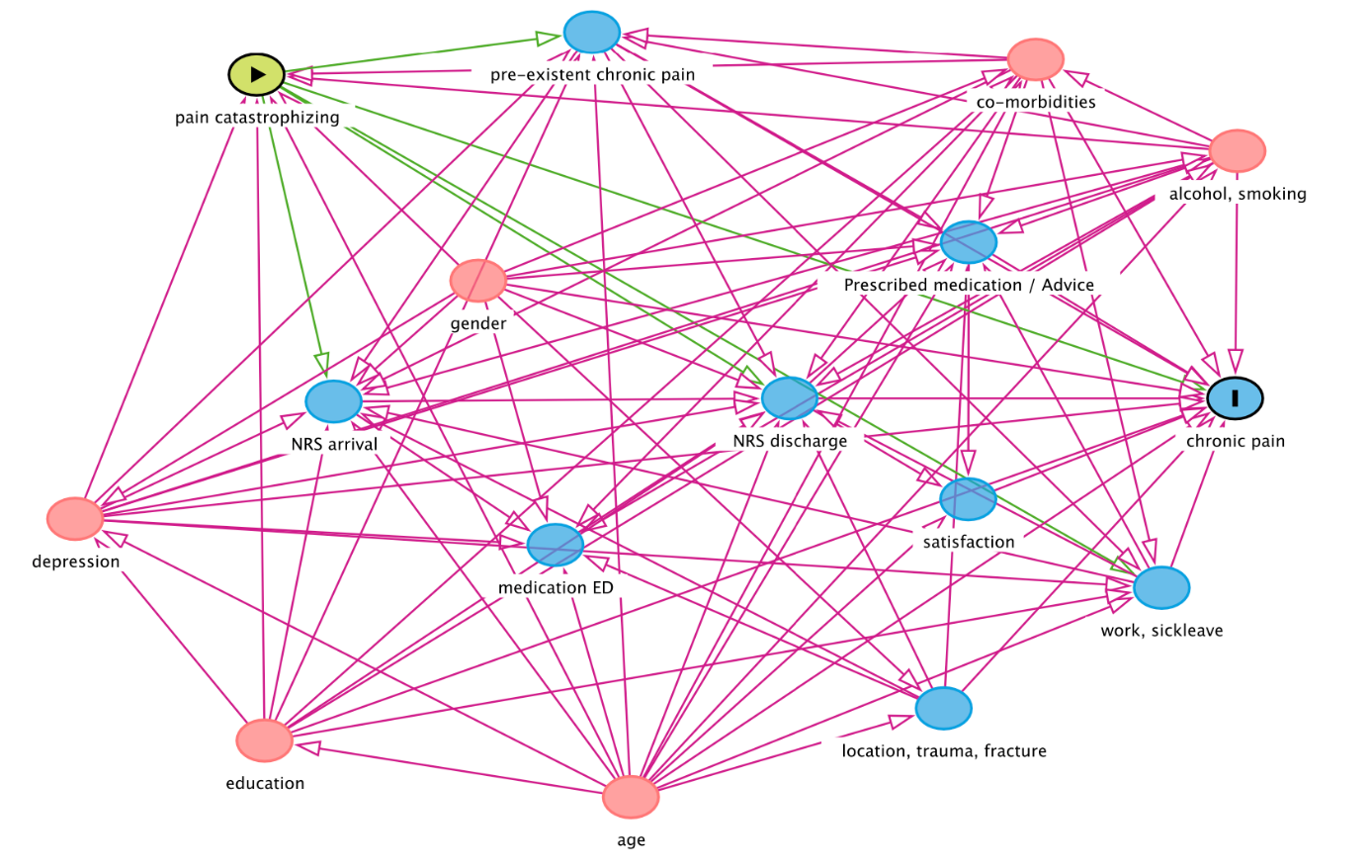


Causal directed acyclic graph on potential confounders of pain catastrophizing

Potential confounders were chosen based on a model built on previous literature, clinical reasoning, clinical experience and by drawing a causal directed acyclic graph (DAG). Based on the DAG, the algorithm selects variables for which needs to be corrected to allow for an estimation of the causal effect of the exposure.
