# Supplementary material for: Sex differences in pain catastrophizing and its relation to the transition from acute pain to chronic pain
Source: BMC Anesthesiol. 2024 Apr 2;24:127. doi: 10.1186/s12871-024-02496-8 (PMC10985981; doi:10.1186/s12871-024-02496-8)
Supplement: Supplementary file 2 — Supplementary Material 2 [file 12871_2024_2496_MOESM2_ESM.docx]

Supplemental table 1. Questions and questionnaires of PRACTICE study.

| Day | NRS of pain | Patient characteristics | EQ-5D-5L | SF-36 |
| --- | --- | --- | --- | --- |
| ED-visit (0) | √ | √ |  |  |
| 1 - 6 | √ | √ |  |  |
| 7 | √ |  | √ |  |
| 90 | √ |  | √ | √ |
| 180 | √ |  | √ | √ |

Schedule for study measurements.

Abbreviations:

NRS: numerating rating scale

SF-36: RAND 36-item Short Form Survey

EQ-5D-5L: Euroqol-5D-5L
