# Supplementary material for: Sex differences in pain catastrophizing and its relation to the transition from acute pain to chronic pain
Source: BMC Anesthesiol. 2024 Apr 2;24:127. doi: 10.1186/s12871-024-02496-8 (PMC10985981; doi:10.1186/s12871-024-02496-8)
Supplement: Supplementary file 3 — Supplementary Material 3 [file 12871_2024_2496_MOESM3_ESM.docx]

Supplemental Table 2: Specification of regression models used for multiple imputation with the mice package in R

|  | | | Covariates used for imputation | | | | | | | | | | | | | | | | | | | | | | | | | | |
| --- | --- | --- | --- | --- | --- | --- | --- | --- | --- | --- | --- | --- | --- | --- | --- | --- | --- | --- | --- | --- | --- | --- | --- | --- | --- | --- | --- | --- | --- |
| Imputed variables | Number of missings, n (%) | Method | Age | Location | Trauma | Fracture | Satisfaction | Depression+Treatment | Depression | Depression treatment | Relationship | Chronic pain | Average alcohol | Alcohol | Education | Work+Sick leave | Work | Sick leave | Smoking | PCS | NRS0 | NRS90 | Pain chronification | Sex | PCS.Age | PCS.Sex | PCS.Depression | PCS.Deprtreatment |  |
| Age | 16 (0.8) | norm |  | v | v |  | v | v |  |  | v | v | v | v | v | v |  |  | v | v | v | v | v | v |  | v | v | v |  |
| Location | 7 (0.4) | logreg | v |  | v |  | v |  |  |  | v | v | v | v | v | v |  |  | v | v | v | v | v | v | v | v | v | v |  |
| Trauma | 628 (33.1) | logreg | v | v |  | v | v | v |  |  | v | v | v | v | v | v |  |  | v | v | v | v | v | v | v | v | v | v |  |
| Fracture | 6 (0.3) | logreg | v | v | v |  | v | v |  |  | v | v | v | v | v | v |  |  | v | v | v | v | v | v | v | v | v | v |  |
| Satisfaction with care received | 882 (46.5) | pmm | v | v | v | v |  | v |  |  | v | v | v | v | v | v |  |  | v |  | v | v | v | v | v | v | v | v |  |
| Depression+Treatment | 751 (39.6) | polyreg | v | v | v | v | v |  |  |  | v | v | v | v | v | v |  |  | v | v | v | v | v | v | v | v |  |  |  |
| Depression | 751 (39.6) | pass | v | v | v | v | v | v |  |  | v | v | v | v | v | v |  |  | v | v | v | v | v | v | v | v |  |  |  |
| Depression treatment | 751 (39.6) | pass | v | v | v | v | v | v |  |  | v | v | v | v | v | v |  |  | v | v | v | v | v | v | v | v |  |  |  |
| Relationship | 744 (39.2) | logreg | v | v | v | v | v | v |  |  |  | v | v | v | v | v |  |  | v | v | v | v | v | v | v | v | v | v |  |
| Chronic pain | 795 (41.9) | logreg | v | v | v | v | v | v |  |  | v |  | v | v | v | v |  |  | v | v | v | v | v | v | v | v | v | v |  |
| Average alcohol | 784 (41.4) | pmm | v | v | v | v | v | v |  |  | v | v |  |  | v | v |  |  | v | v | v | v | v | v | v | v | v | v |  |
| Alcohol | 784 (41.4) | pass | v | v | v | v | v | v |  |  | v | v | v |  | v | v |  |  | v | v | v | v | v | v | v | v | v | v |  |
| Education | 822 (43.4) | polyreg | v | v | v | v | v | v |  |  | v | v | v | v |  | v |  |  | v | v | v | v | v | v | v | v | v | v |  |
| Work+Sick leave | 819 (43.2) | polyreg | v | v | v | v | v | v |  |  | v | v | v | v | v |  |  |  | v | v | v | v | v | v | v | v | v | v |  |
| Work | 819 (43.2) | pass | v | v | v | v | v | v |  |  | v | v | v | v | v | v |  |  | v | v | v | v | v | v | v | v | v | v |  |
| Sick leave | 819 (43.2) | pass | v | v | v | v | v | v |  |  | v | v | v | v | v | v |  |  | v | v | v | v | v | v | v | v | v | v |  |
| Smoking | 887 (46.8) | logreg | v | v | v | v | v | v |  |  | v | v | v | v | v | v |  |  |  | v | v | v | v | v | v | v | v | v |  |
| PCS | 943 (48.3) | pmm | v | v | v | v | v | v |  |  | v | v | v | v | v | v |  |  | v |  | v | v | v | v |  |  |  |  |  |
| NRS0 | 5 (0.3) | pmm | v | v | v | v | v | v |  |  | v | v | v | v | v | v |  |  | v | v |  | v | v | v | v | v | v | v |  |
| NRS90 | 1,077 (56.8) | pmm | v | v | v | v | v | v |  |  | v | v | v | v | v | v |  |  | v | v | v |  |  | v | v | v | v | v |  |
| Pain chronification | 1,077 (56.8) | pass | v | v | v | v | v | v |  |  | v | v | v | v | v | v |  |  | v | v | v | v |  | v | v | v | v | v |  |
| Sex | 6 (0.3) | logreg | v | v | v | v | v | v |  |  | v | v | v | v | v | v |  |  | v | v | v | v | v |  | v |  | v | v |  |
| PCS.Age | 899 (47.4) | pass | v | v | v | v | v | v |  |  | v | v | v | v | v | v |  |  | v | v | v | v | v | v |  | v | v | v |  |
| PCS.Sex | 893 (47.1) | pass | v | v | v | v | v | v |  |  | v | v | v | v | v | v |  |  | v | v | v | v | v | v | v |  | v | v |  |
| PCS.Depression | 900 (47.5) | pass | v | v | v | v | v | v |  |  | v | v | v | v | v | v |  |  | v | v | v | v | v | v | v | v |  | v |  |
| PCS.Deprtreatment | 900 (47.5) | pass | v | v | v | v | v | v |  |  | v | v | v | v | v | v |  |  | v | v | v | v | v | v | v | v | v |  |  |

Specification for multiple imputation. The left column displays the imputed variables. In the row it states which variables were used to calculate the imputed variable.

Abbreviations:

V: variable used to correct data imputation

logreg: Logistic regression
norm: Bayesian linear regression
pass: Passive imputation (weight / length^2^)
pmm: Predictive mean matching

polyreg: Polytomous regression
