# Supplementary material for: Sex differences in pain catastrophizing and its relation to the transition from acute pain to chronic pain
Source: BMC Anesthesiol. 2024 Apr 2;24:127. doi: 10.1186/s12871-024-02496-8 (PMC10985981; doi:10.1186/s12871-024-02496-8)
Supplement: Supplementary file 4 — Supplementary Material 4 [file 12871_2024_2496_MOESM4_ESM.docx]

Supplemental table 3: Baseline characteristics of patients with and without missing data

| **Study variables** | **PCS complete** | **PCS missing** | **P-value** |
| --- | --- | --- | --- |
| **Male**, n (%) | 472 (46.9) | 501 (56.1) | <0.001*** |
| **Age**, median (IQR) | 47 (31 - 59) | 43 (27 - 58) | 0.001** |
| mean (SD) | 45.9 (16.8) | 43.6 (17.6) | 0.005** |
| [n] | [1001] | [889] |  |
| **NRS0**, median (IQR) | 5.0 (3 - 7) | 5.0 (3 - 7) | 0.361 |
| mean (SD) | 4.9 (2.6) | 5.0 (2.6) | 0.363 |
| [n] | [1009] | [892] |  |
| **Location**, n (%) |  |  |  |
| Abdomen | 58 (5.8) | 59 (6.6) | 0.445 |
| Upper extremities | 495 (49.2) | 437 (49.0) | 0.926 |
| Cervical spine | 20 (2.0) | 23 (2.6) | 0.392 |
| Head | 77 (7.7) | 93 (10.4) | 0.036* |
| Lower extremities | 368 (36.6) | 289 (32.4) | 0.056 |
| Thorax | 63 (6.3) | 72 (8.1) | 0.129 |
| Thoracic and lumbar spine | 31 (3.1) | 29 (3.3) | 0.833 |
| **Trauma**, n (%) | 605 (87.8) | 493 (83.7) | 0.037* |
| **Fracture**, n (%) | 581 (57.7) | 405 (45.4) | <0.001*** |
| **Satisfaction with care received**, median (IQR) | 8 (7 - 9) | 8 (7 - 8) | 0.033* |
| mean (SD) | 7.7 (1.6) | 7.4 (2.0) | 0.033* |
| [n] | [854] | [164] |  |
| **Depression**, n (%) | 194 (19.4) | 25 (16.8) | 0.430 |
| **Treatment**, n (%) | 54 (27.8) | 7 (28.0) | 0.987 |
| **Relationship** | 705 (70.0) | 94 (63.1) | 0.103 |
| **Chronic pain in other location**, n (%) | 231 (23.3) | 21 (18.6) | 0.232 |
| **Alcohol consumption**, n (%) | 487 (48.6) | 53 (46.5) | 0.670 |
| **Consumption per week**, median (IQR) | 0 (0 - 1) | 0 (0 - 1) | 0.669 |
| mean (SD) | 0.5 (0.5) | 0.5 (0.5) | 0.670 |
| [n] | [1002] | [114] |  |
| **Education** |  |  |  |
| None | 14 (1.4) | 0 (0.0) | <0.001*** |
| Low | 23 (2.3) | 5 (6.6) | 0.144 |
| Intermediate | 546 (54.5) | 38 (50.0) | 0.455 |
| High | 419 (41.8) | 33 (43.4) | 0.787 |
| **Employment** | 775 (77.1) | 55 (72.4) | 0.376 |
| Sickleave | 385 (49.7) | 16 (29.1) | 0.002** |
| **Smoking**, n (%) | 158 (16.0) | 5 (18.5) | 0.744 |

Comparison between baseline characteristics of patients with and without missing data on PCS.

NRS: Verbal Numeric Rating Scale, NRS90: Numeric Rating Scale at day 90, PCS: Pain Catastrophizing Scale, n: Number of samples, IQR: Interquartile range, SD: Standard deviation

*p<0.05, **p<0.01, ***p<0.001, based on student's t-tests, Wilcoxon-Mann-Whitney tests, and ^c^ chi-squared tests

Low level of education: primary school, Pre-vocational secondary education, Secondary vocational education level 1 Or completion of the first three years of Senior general secondary education or Pre-university education

Intermediate level of education: graduation on senior general secondary education, pre-university education, secondary vocational education level 2-4

High level of education: Graduation at least university of applied sciences
